# Ideology and free speech values predict content moderation preferences: cross-national evidence across targets of hate speech

**DOI:** 10.1038/s41598-026-56054-y

**Published:** 2026-06-17

**Authors:** Yannis Theocharis, Spyros Kosmidis, Jan Zilinsky, Friederike Quint

**Affiliations:** 1 Department of Governance, Munich School of Politics and Public Policy, Technical University of Munich, Munich, Germany; 2 Department of Politics and International Relations, University of Oxford, Oxford, UK; 3 Centre of Social and Psychological Sciences, Slovak Academy of Sciences, Bratislava, Slovakia

**Keywords:** Social media, Content moderation, Freedom of speech, Politics and international relations, Psychology, Psychology, Science, technology and society

## Abstract

With over 4 billion people using social media platforms daily, decisions about what content to allow or moderate have profound implications for global public discourse, democratic deliberation, and users’ psychological well-being. This study examines cross-national variation in content moderation preferences by exposing participants in 10 countries to a standardized example of hate speech: a hateful post modeled on text that X (formerly Twitter) had cited in its content moderation guidelines. Across contexts, exposure to such content triggers widespread support for intervention, with minimal differences based on the experimentally varied identity of the target. However, significant variation emerges across countries. The United States stands out for its comparatively lower support for moderation of hate speech, while countries like France, Brazil, and South Africa exhibit much stronger demand. Within most countries, political ideology predicts support: individuals on the right are less likely to endorse removal or suspension than those on the left. Crucially, values around freedom of speech versus harm prevention consistently shape preferences across all contexts. Those who prioritize prevention from harm are markedly more likely to support hard forms of moderation (e.g., account suspension and content take-downs). These findings highlight that moderation preferences are shaped less by the specific target than by individuals’ deeper commitments regarding free expression and harm prevention.

## Introduction

Social media platforms have long been celebrated as powerful tools for personal and political expression. More recently, however, they have increasingly been criticized for their role in amplifying misinformation and hate speech^1,2^. As these platforms continue to grow^3^, the challenge of balancing free expression with the need to protect users from harm has become more urgent^4,5^. The debate over content moderation—what to allow, what to remove, and who decides—has sparked polarized discussions worldwide^6–10^. In the United States (US), the debate has become so contentious that the Trump administration used economic pressure to defend American free speech norms abroad, warning the UK during trade talks that its hate speech laws could harm US platform profits and weaken the UK’s negotiating position, and threatening Brazil with a 50% tariff over court-ordered content removals targeting US social media companies^11,12^.

### The global stakes of platform governance and the influence of users as moderators

While digital technology companies, civil society organizations, and policymakers engage in these debates, the perspectives of users are largely overlooked. Yet, users have always played an integral role in content moderation. Early efforts, such as Facebook’s content flagging system, relied heavily on user reports to identify harmful material, reflecting an early crowdsourced approach to moderation^13^. Similarly, Twitter’s Birdwatch program, which was launched in 2021 and was later rebranded as Community Notes under X, further brought user participation into content moderation. The expanded role of community notes along with Meta’s removal of third-party fact-checking partnerships^14^, have renewed emphasis on user-centered moderation as a prominent strategy after years of prioritizing automation^15^. Meta’s decision to end fact-checking in the US was accompanied by Zuckerberg’s explicit statement that the company’s apps would shift greater responsibility to users by relying more on user reporting for some types of violations^16^. Beyond direct reporting, users also act as moderators through counter speech, directly challenging hateful or misleading content. For example,  Siegel and Badaan^17^ showed in a field experiment in Lebanon that counter speech reduced sectarian hate speech online, while Hangartner and colleagues^18^ found that counter speech interventions in Germany lowered xenophobic hate and improved the tone of online discussions. Along with counter-speech, users influence content moderation and the health (respect, openness, tolerance, civility) of the broader social media environment in significant ways through their behavior on platforms. Algorithms prioritize content that garners high engagement, often including misleading or hateful material^19^, while user behaviors, such as liking or sharing such content, further amplify it^20^. In this way, both algorithmic design and user preferences contribute to what content is promoted or suppressed^21^. However, the precise influence of each remains difficult to assess due to the opacity of algorithmic decision-making.

Understanding user preferences around content moderation is crucial, not only because they reveal how citizens navigate the tension between free speech and harm prevention, but also because they shape how platforms govern online content and discourse. In the US, public debate often frames moderation as a binary between liberty and censorship. For example, Elon Musk has characterized content moderation as “a propaganda word for censorship”^22^. This perspective, currently embraced by the political right, obscures a deeper normative conflict: the challenge of balancing the protection of expression with the imperative to shield individuals from hate speech and harassment which can suppress participation and undermine democratic equality^23^.

But it remains unclear whether the ideological associations with free speech observed in the US hold in other national contexts. This uncertainty is especially important given that, historically, appeals to free speech have been made across the ideological spectrum—including by the left, liberals, and conservatives. This, therefore, invites a more systematic examination of how left–right ideology and values related to free speech may shape user preferences in diverse political and cultural settings.

These preferences, in turn, play a critical role in shaping platform strategies. While legal and ethical considerations remain important, moderation decisions are frequently guided by economic incentives. Platforms design their policies to retain users, protect their reputation, and maximize engagement^7,21^. Insufficient moderation can alienate users, while overly aggressive moderation risks backlash and accusations of bias. As a result, most platforms operate in a precarious balance between profitability, public trust, and an evolving sense of corporate responsibility^24^.

### Free speech, harm, and the limits of universal norms

Adding further complexity to this balancing act is the global nature of social media platforms. Most of the platforms dominating the social media market, where much of political speech online is happening (Facebook, YouTube, Instagram, X), are American companies. Legal analysis has shown that their content moderation policies have been shaped by American legal norms and cultural values, reflecting the perspectives of US-based engineers and product teams^24^. However, users in the US account for only a very small share of the global social media population. As these platforms continue to expand internationally^3^, the question of what constitutes acceptable speech—and who gets to decide—becomes more complicated.

One reason cross-national comparisons of content moderation are theoretically important is that free speech cultures differ substantially across legal and political contexts. While the American model emphasizes liberty, individual autonomy, and skepticism toward government restrictions on speech, many European systems place greater weight on dignity, civility, and collective harmony^25^. These differences are reflected in distinct legal and regulatory approaches. Germany’s postwar Basic Law places dignity at its core, helping to justify strict bans on hate speech, Holocaust denial, and personal insult even in the absence of imminent harm^26^. France similarly restricts speech that undermines republican values^27^, while the UK criminalizes threatening or abusive speech under the Public Order Act 1986 and the Communications Act 2003, with the Online Safety Act of 2023 extending regulatory oversight of platform governance. At the European level, the Digital Services Act requires very large platforms to assess and mitigate systemic risks such as disinformation and hate speech^28^. These approaches contrast with the US framework, where even false or offensive speech receives broad constitutional protection, and where Section 230 of the 1996 Communications Decency Act grants platforms wide discretion, but not a general obligation, to moderate harmful content^29^. This legal insulation, combined with a cultural reverence for expressive freedom, makes the US an outlier not only in law^25^ but also in how it influences global platform norms. As international standards increasingly emphasize harm reduction, these discrepancies generate friction. Moderation policies deemed necessary in one country may appear as censorship in another, turning content governance into a geopolitical challenge shaped by the tension between global reach and local legitimacy.

These legal regimes provide important context for the broader liberty–dignity divide that motivates our comparative design. If citizens’ moderation preferences are shaped primarily by national legal and cultural traditions, attitudes should vary systematically across these contexts. If, instead, preferences are rooted in individual-level commitments regarding free expression and harm prevention, similar value-based patterns should emerge even across countries with different legal traditions. This study therefore asks whether citizens’ moderation preferences track divergent legal traditions or reflect deeper value commitments that cut across national contexts.

### Past work

Empirical research on users’ attitudes towards content moderation is growing, yet there is still limited evidence from a global, comparative perspective. Existing work has largely focused on users’ preferences regarding the targets of hateful speech and disagreements over whether misinformation should be removed, which often depends on who is sharing it. Most of this research has focused on the US^30–32^, though some scholars have also looked at Germany and Denmark^33,34^, offering valuable insights but yielding mixed findings on the relative importance of target identity in shaping support for content moderation.

Some studies suggest that citizens’ attitudes are primarily driven by the severity of the language used. Rasmussen, for example, finds that individuals across partisan lines are more likely to support regulation of hate speech when the content is extreme or threatening, regardless of who is being targeted^34^. In contrast, other studies suggest that target identity plays a meaningful role. Using a broader set of social groups, Pradel et al. report that threats directed at LGBTQ individuals elicit higher support for moderation than similar threats against other groups^32^. Solomon et al., meanwhile, find broad cross-partisan agreement on which groups should be prioritized for protection from hate speech^30^. In their study, Democrats and Republicans differ more in the amount of ‘censorship’ they prefer, rather than in who they believe deserves protection. Another recent study also shows that Republicans’ posts are more likely than Democrats’ to be labeled as misleading in community notes, revealing an asymmetry in the sharing of misinformation^35^.

Motivated reasoning as a driver of (in)tolerance toward content posted by specific groups is also a central focus in this literature—and one marked by mixed findings. Several studies find that people do not consistently favor their own political or social in-groups when evaluating harmful content. Pradel et al. report minimal evidence of partisan-motivated reasoning, showing that Democrats and Republicans do not selectively support moderation based on group membership^32^. Similarly, Solomon et al.^30^ find broad cross-partisan agreement on which groups (particularly Black and Jewish individuals) deserve protection from hate speech, with little evidence that partisans favor speech targeting their political out-groups. Kozyreva et al. also show that users focus more on the potential harm and repetition of misinformation than on the identity of those spreading it^36^. Rasmussen’s finding that partisans across the spectrum support restricting extreme speech regardless of the targeted group, also emphasizes severity over group affiliation^34^. In contrast, Munzert et al. detect in-group biases: in their study, Americans were more tolerant of hateful speech from their ideological in-group and more protective when their in-group was targeted^33^. Appel et al.^31^ further highlight that although Republicans and Democrats differ in their content moderation preferences, these differences are not primarily driven by in-group favoritism, but by deeper ideological commitments.

The present study extends this literature in three ways. First, while existing work remains largely US-centered or limited to a small set of specific cases, we provide cross-national evidence from 10 countries spanning liberty-based and dignity-based legal traditions, allowing us to assess whether findings from this literature travel across diverse legal, political, and cultural contexts. Second, by exposing respondents to a standardized hate speech stimulus while experimentally varying only the target’s identity, we estimate the causal effect of target identity on preferences for no, soft, and hard moderation, testing whether citizens apply consistent standards to hateful content regardless of who is attacked. Third, we move beyond political ideology by directly measuring values related to free speech and harm prevention, showing that these values consistently predict hard moderation preferences across national contexts. Together, these contributions shift the focus from whether users support moderation in a single national setting to when, for whom, and on what normative basis citizens across democracies endorse platform intervention.

## Research design

Our analysis offers a distinctive global perspective on how citizens navigate the trade-off between protecting free expression and preventing harm. It presents cross-national data from 10 countries (N = 13,475), with similar sample sizes across all countries and broadly comparable demographic profiles in terms of gender, age, and education (see Table 1)^1^ with diverse historical experiences in limiting free speech (Greece, Germany, Brazil and Slovakia all experienced authoritarian rule in their modern history), as well as with diverse political, cultural, and legal contexts. Our selection includes several paradigmatic cases of dignity-based (France, South Africa, Germany, Brazil) and liberty-based (US, Sweden) legal systems, where public attitudes towards content moderation may differ based on the underlying normative frameworks that emphasize either protection from harm or individual freedom of expression.

This variation is analytically consequential: if attitudes toward content moderation were simply artifacts of local legal environments, we would expect preferences to track national legal traditions closely. By sampling across both ends of the liberty–dignity spectrum—and including countries that do not fit neatly into either category, such as Greece, Slovakia, and Australia—we can distinguish whether citizens’ moderation preferences reflect the legal norms they have been socialized into or deeper individual-level value commitments that operate independently of institutional context.

**Table 1:** Sample demographics by country (weighted estimates).

**Country**	**N**	**% Female**	**Median age**	**% High Edu.**
Australia	1308	50.1	42	37.8
Brazil	1364	49.1	40	17.2
France	1380	47.6	45	23.4
Germany	1353	48.9	46	28.7
Greece	1381	49.3	46	29.3
Slovakia	1379	48.8	44	26.2
South Africa	1324	49.0	36	11.1
Sweden	1348	49.7	43	34.3
USA	1290	50.3	42	39.5
United Kingdom	1348	50.7	43	39.9
Total	13475	49.3	43	28.7

Participants completed an online survey which was structured as follows: first, respondents provided sociodemographic information, including their age, gender, and level of education. Subsequently, they indicated their political orientation using an ideological self-placement scale. The survey then presented a series of questions regarding their online experiences, followed by a key question designed to assess their core values, which asked them to state whether they prioritized free speech over protection from harm. After a wash out period, where they answered other attitudinal items, we embedded an experiment where respondents viewed a social media post in which the identity of an attacker’s target was randomly manipulated. Placing this experimental manipulation at the end was an essential design choice to prevent post-treatment bias, ensuring that all covariate measurements were captured before any treatment exposure.

We build on previous research by examining the underlying value commitments that shape attitudes toward content moderation, particularly the influence of political ideology and support for free speech^31^. We also focus on hate speech rooted in group-based discrimination, as responses to this type of abuse may differ across cultural and historical contexts^37^. Following prior work showing that more severe language produces the strongest evaluative reactions^32^, we used an unambiguously hateful stimulus to establish a clear baseline: if cross-national and individual-level differences emerge even in response to an extreme case, they are likely to be robust.^2^ To ensure validity, the stimulus was intentionally designed to be both comparable and decontextualized. Because meaningful comparison required language interpretable across all ten countries, we avoided culture-specific idioms, slang, or references (e.g., terms that would be salient in the US political discourse but meaningless or misleading in Greece). With this in mind, we worded our treatment after an example provided by X (Twitter) in its own content moderation guidelines to illustrate a group-directed hate speech policy violation: “[Religious Group] should be punished. We are not doing enough to rid us of those filthy animals”^38^. This decontextualized approach was also essential for isolating general attitudes toward hateful and dehumanizing speech, while avoiding reactions tied to any particular country’s policies, controversies, recent events, or to perpetrators who could be construed as either in-groups or out-groups. This approach aligns with the treatment design strategies adopted by others^32,33,36^, who similarly use standardized vignettes to measure public reactions to norm-violating content.^3^

To ensure that respondents were exposed to more than one target of hate, we experimentally varied the identity of the group being targeted: (a) a person of a different skin color or ethnic group^4^, (b) a person of a different gender, or (c) a person of a different sexual orientation than the attacker. Each respondent was randomly assigned to view only one version of the post, targeting either gender, race/ethnic group, or sexual orientation. The text was translated into the primary language of each country by professional translators and subsequently verified by academic experts native to those contexts. It read as follows:

Imagine someone sends this social media post attacking a person because they have a different **skin color or belong to a different ethnic group** **[gender]** **[sexual orientation]** than the attacker:

“They should be punished! We are not doing enough to rid us of those filthy animals.”

After reading the post, respondents were asked—again in similar fashion to other work^30,32^—how they believed platforms like Facebook or X should respond. The response options were:

*Leave it, do nothing; Place a warning label on the post; Reduce how many people can see the post; Permanently remove the post; Suspend the person’s account.*

For the analysis, we grouped these responses into three categories: **no moderation** (*Leave it, do nothing*), **soft moderation** (*Place a warning label on the post; Reduce how many people can see the post*), and **hard moderation** (*Permanently remove the post; Suspend the person’s account*).

By holding the severity and type of hateful speech constant while varying only the identity of the target, our experimental design isolates the causal effect of target identity on respondents’ support for content moderation. This allows us to assess whether users apply consistent standards to hateful content, or whether their judgments vary depending on who is being attacked. Unlike observational approaches, which risk confounding message tone, target identity, and platform context, our method ensures that all participants evaluate the exact same language (involving a clear violation of norms of civility and non-violence) thereby providing a clean test of group-based bias in moderation preferences.

Importantly, this approach improves on more abstract survey questions about norms or values, which may elicit socially desirable responses or reflect broad principles that respondents might not apply in practice. By confronting participants with a concrete and clearly hateful post, the experiment captures real-time reactions to hate speech that more closely mirror decisions users and platforms must make. The design thus allows us to measure behaviorally relevant attitudes about content moderation in a way that is both internally valid and directly policy-relevant.

## Results

### Variation across countries

**Fig. 1:**
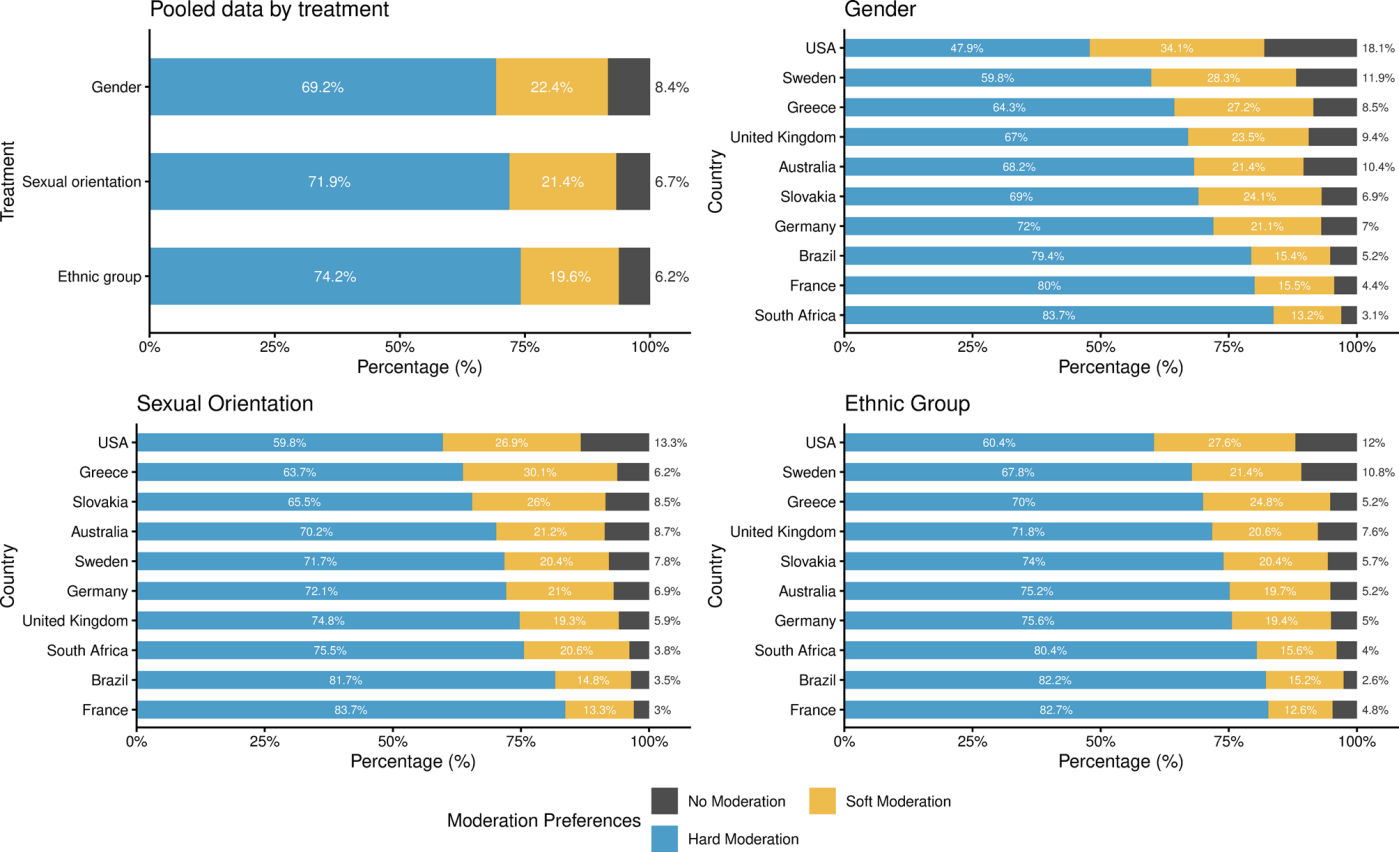
Moderation preferences across countries and targets, Top left, target effects pooled across countries. Country-specific variation in moderation preferences when someone is targeted because of their gender (top right), sexual orientation (bottom left), ethnic origin/race (bottom right). Colors indicate the type of moderation action preferred: hard moderation, soft moderation, or no moderation.

We begin by examining how respondents across 10 countries react to the hateful post, and how many call for intervention. Across our full sample, nearly three-quarters (72.4%) support hard moderation, such as post removal or account suspension, while only 6.9% believe platforms should do nothing.^5^ The remaining 21.1% prefer softer interventions, such as warning labels or visibility reduction. Since hard moderation, such as banning users or taking down content, represents by far the strongest form of user response to hateful speech—and is also the form of moderation most frequently cited in public debates about censorship on social media (see, for example, reporting by Kang^39^)—it serves as the primary quantity of interest in our analysis. We therefore focus on this outcome in the results that follow, while also reporting figures for soft and no moderation to capture the full spectrum of preferences. Figure 1  visualizes moderation preferences pooled across countries for each target group (top-left panel) and pooled across target groups for each country (remaining panels). In general, the differences across our targets are not large. When the hateful post targets someone’s gender, 69.2% call for hard moderation—slightly lower than when the hateful content targets one’s ethnic origin (74.2%) or sexual orientation (71.9%).^6^ However, this apparent consistency in the pooled sample masks important cross-national variation. The country-specific panels in Fig. 1 show that both the level of support for moderation and in some cases the sensitivity to target identity vary considerably by country.^7^

The top right panel of Fig. 1 reveals important cross-national differences in how respondents react when a person is attacked because of their gender. Support for hard moderation ranges from just 47.9% in the US all the way up to 83.7% in South Africa. The US stands out for its relatively low demand for any action: nearly 1 in 5 Americans (18.1%) consider the post appropriate for social media. By contrast, in South Africa, fewer than 4% express this view.

The results for the gender condition represent the lowest support for moderation across all three target types. When examining the sexual orientation condition (bottom left in Fig. 1), support for hard moderation rises to 59.8% in the US. This represents a statistically significant increase of about 12 percentage points compared to the gender condition, and yet the US once again ranks at the bottom end of the distribution. At the other end of the distribution, support for hard moderation climbs to 83.7% in France and 81.7% in Brazil. This is comparable with what we see in the bottom right of Fig. 1, which visualizes moderation preferences in the ethnic group treatment, with Brazil, France, and South Africa showing consistently high support for strong moderation and the US lagging behind. Looking across all conditions, we see that, irrespective of the target, 14.4% of Americans express no support for any moderation of the hateful post at all—the highest figure in our sample. At the other end of the spectrum, only around 3–4% of respondents in South Africa and Brazil reject platform intervention. These contrasts are mirrored in the overall levels of support for hard moderation, which range from 56.1% in the US to over 81% in Brazil and 82.1% in France.^8^

### Variation within countries

An important question is whether there is anything approaching a societal consensus on how harmful content should be handled, or whether attitudes toward moderation diverge sharply *within* countries. To explore this, we focus on our main outcome of interest: support for hard moderation. First, we examine how this preference varies by political ideology—which we measure with the ‘liberal’ vs ‘conservative’ (for the US) and 'left vs. right’ scales for the rest—pooling responses across all three target groups. We note here that while the substantive content of ‘left’ and ‘right’ varies across contexts, comparative politics research shows that these labels consistently structure political attitudes across all the democracies we study.^9^ Ideological labels are meaningful social constructs that have historically oriented people’s political choices, and recent research confirms their relevance, demonstrating that these labels are rooted in stable individual differences in personality and core values, which in turn predict political preferences^40,41^.

**Fig. 2:**
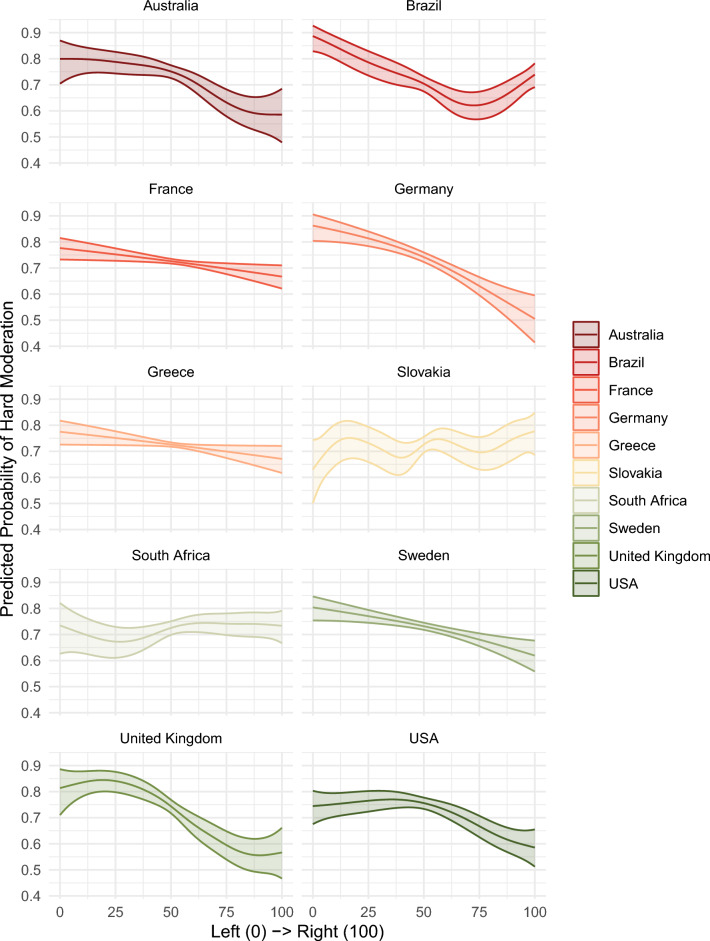
Predicted probabilities with 95% confidence intervals of preferring hard moderation across countries by left-right ideology. Each panel shows estimates for a different country, with colored lines indicating the predicted probability of preferring hard moderation and shaded areas representing 95% confidence intervals. Estimates are smoothed using Generalized Additive Models to capture potential non-linear relationships between ideology and moderation preferences. The U.S. ideology scale ranges from 0 = “Liberal” to 100 = “Conservative”.

Figure 2 displays the predicted probability of endorsing hard moderation across the full 0–100 ideological spectrum. With the exception of the United States, where content moderation has been studied extensively and is highly politicized, we remain agnostic about cross-national associations. To explore these dynamics, we estimate predicted probabilities using Generalized Additive Models (GAMs^42^), which flexibly capture nonlinear relationships (e.g., the possibility that individuals at the ideological extremes behave similarly) without imposing a specific functional form on the data. This approach–widely used in political science^43,44^–enables us to identify the regions of the ideology scale where the probability of supporting hard moderation shifts most clearly.^10^

The results reveal a broadly consistent ideological pattern across most countries: right-leaning respondents are less likely to support hard moderation than those on the left. In countries like France, Greece and Germany, this relationship is remarkably linear, with support for moderation gradually declining as we move from the left to the right (see Table A6 in the supplementary materials for the relevant statistics). Notably, in these contexts, support remains relatively high across the spectrum—meaning that while those on the left are generally more supportive, even respondents on the right frequently endorse content removal or account suspension. However, the contrast is strongest in Germany, where support for hard moderation declines sharply as the shift to the right progresses. Two countries stand out as exceptions to this pattern. In both South Africa and Slovakia, support for hard moderation remains relatively flat across the ideological spectrum. Meanwhile, Australia, the UK and the US exhibit a nonlinear pattern: support for hard moderation is high for those left of center, but it drops off sharply as respondents move from the center toward the conservative/extreme right. When calculating within-country 90th (extreme right) to 10th percentile (extreme left) effects, parametric results show that with the exception of South Africa and Slovakia, where the differences in the probability of preferring hard moderation are indistinguishable from 0, there are significant ideological differences. The left-right gap can range from 14% in France to 24% in the UK (full results in Table A4 in the supplementary materials). Finally, Figure A6 in the supplementary materials reports the same effects across targets.^11^

While ideology reflects a broad set of views about government and society, attitudes toward freedom of speech potentially represent the most direct link to content moderation preferences. This is precisely why examining ideology alongside free speech values is essential: if ideology were the sole driver, countries like Slovakia and South Africa–where left–right placement has little bearing–would appear to lack meaningful attitudinal structure altogether. As we show below, free speech values reveal a consistent cleavage that ideology alone obscures.

**Fig. 3:**
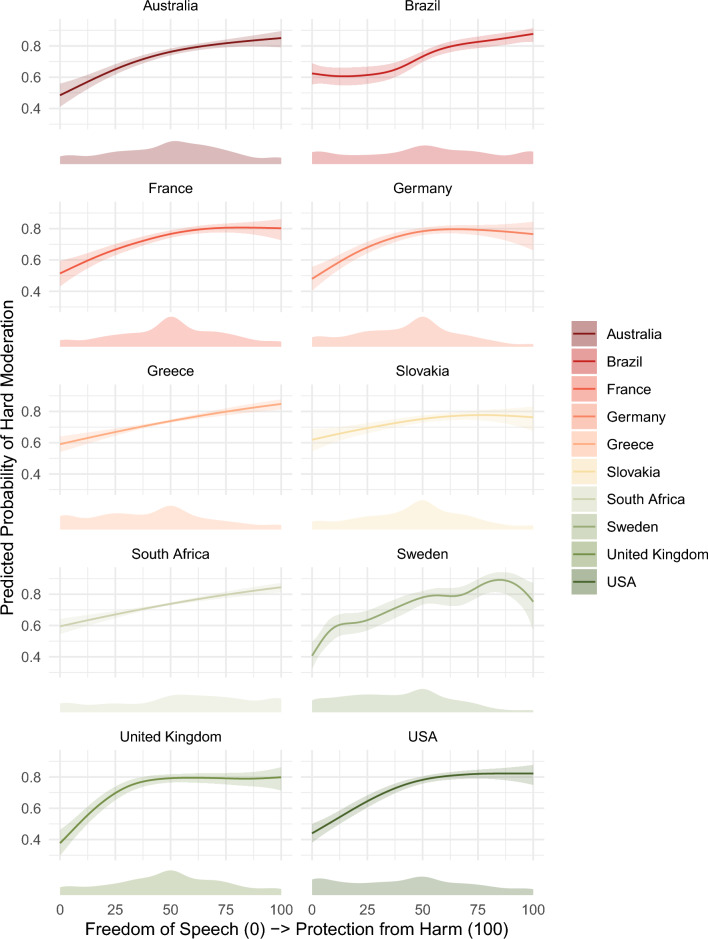
Predicted probabilities with 95% confidence intervals of preferring hard moderation across countries by freedom of speech preferences. Each panel shows estimates for a different country. Colored lines indicate the predicted probability of preferring hard moderation, and shaded areas represent 95% confidence intervals. Estimates are smoothed using semiparametric Generalized Additive Models to capture potential non-linear relationships between freedom of speech preferences and moderation preferences. The small density plots along the x-axis display the distribution of respondents’ free-speech versus harm-prevention values.

Freedom of speech has been central to debates over platform governance and, as we discussed earlier, represents a major dividing line between liberty-centric and dignity-centric traditions of expression. To assess how citizens respond to this tradeoff in light of these traditions, we asked respondents the following question prior to treatment exposure: *In general, how important is freedom of speech relative to the harm it might cause?*, with 0 indicating strong preference for free speech and 100 indicating strong preference for protection from harm. We then examined how placement on this scale predicts support for hard moderation, using the same nonparametric smoothing approach applied to ideology.^42^ The results, shown in Fig. 3, reveal a consistent pattern across countries indicating that freedom of speech vs protectionist values matter: the more respondents prioritize protection from harm, the more likely they are to support hard forms of moderation. The small density plots along the x-axis illustrate the distribution of respondents’ positions on this value scale. Unlike political ideology, which varies in its explanatory power, this value-based tradeoff predicts attitudes in all national samples. Even in countries like Slovakia, where ideological self-placement had little bearing, harm-prevention values strongly correlate with support for content removal or account suspension. An inspection of the plots shows that, in most cases, when those values exceed 50 the demand for hard moderation reaches its peak.^12^

When replicating the left–right gap analysis using the free speech trade–off, we observe a much stronger and consistent effect. The 90-10% gap for free speech values ranges from 11% in Slovakia to 30% in Sweden and the US, all statistically significant at conventional levels. The latter set of countries are generally pro-free speech and have the lowest levels of support for moderation. The UK, whose respondents were relatively more demanding of moderation, has a value gap of 25% in the probability of demanding hard moderation. This is comparable to Australia and Brazil (27% and 26%, respectively).^13^

We also examine the joint effects of ideology and free speech values. As before, we estimated these models using both parametric and semiparametric methods and calculated the probability of users’ demands over different types of moderation (no moderation, soft moderation, hard moderation). We considered each variable at their full range of values and then calculated the effects for the mean and ±1SD around the mean for the other variable. When examining ideology at its full range, we find that in the case of soft moderation only those who are strong proponents of free speech seem to be moved by ideology. Even left-leaning free-speech advocates are significantly more likely to have no demands for moderation. When examining the same group’s preferences for hard moderation, the results reveal that ideology still drives the trend at a similar rate, but the decline is even steeper among those with strong free speech preferences. When we hold ideology constant, just as we did for free speech values, the findings show that for the no-moderation scenario, ideology makes no difference among those scoring above 50 on the scale. Likewise, in the hard moderation scenario, regardless of ideological orientation, individuals who prioritize protection from harm over free speech demonstrate strong support for strict moderation. This is generally confirmed when visualizing the results using semiparametric modeling. The above patterns are visualized in Figures A7, A8, and A9 in the supplementary materials.

Finally, we observe additional sources of heterogeneity in moderation preferences. Older respondents tend to be more supportive of hard moderation (see Figure A10, supplementary materials), while heavy social media users are consistently less likely to demand strong intervention. Platform use also matters: individuals who use Facebook are more likely to support hard moderation, whereas X (formerly Twitter) users are less likely to do so. These patterns are detailed in Figure A11 in the supplementary materials.

## Discussion

Contrary to the polarized rhetoric that often dominates public debate and the pronouncements of some tech and policy elites, most social media users do not appear to view content moderation as a binary choice between absolute free speech and censorship. Instead, our findings suggest that when confronted with hateful language, citizens across diverse societies generally support some form of intervention. This support holds even when the targets of hate vary as a result of our experimental manipulation, indicating that it is the extremity of the language, and less so the identity of the group targeted, that primarily drives public demand for moderation. This key finding, which aligns with previous work on content moderation preferences^30,34^, also resonates with core principles in normative democratic theory. Broad-based opposition to hateful speech reflects a shared commitment to maintaining standards of respect and civility in public discourse, particularly when such speech borders on incitement or verbal violence.^45^ Normative theorists, especially those concerned with the protection of vulnerable or marginalized groups, have long argued that unfettered expression can undermine equal participation, silence minority voices, and erode the conditions necessary for inclusive democratic deliberation^46,47^.

Our results highlight two core sources of resistance to content moderation: political ideology and value commitments. In most countries, right-leaning respondents are less likely to support hard moderation in the form of content removal or account suspension than those on the left, reflecting a familiar ideological divide over regulatory intervention. However, this pattern is not universal. In countries such as South Africa and Slovakia, ideological orientation plays little or no role, suggesting that resistance to moderation may sometimes emerge from broader social consensus or nonpartisan norms.

More consistently—and more powerfully across contexts—we find that value commitments related to free speech strongly predict support for content moderation, and this holds also for countries whose legal systems sit at the opposite ends of the liberty–dignity spectrum, such as the US and France or Germany. Across all 10 countries, individuals who prioritize freedom of expression over protection from harm are significantly less likely to support hard moderation, even when confronted with extremely toxic language. While our stimulus necessarily abstracted from the full complexity of online contexts (e.g., account characteristics, irony, slurs, etc.), this choice allowed us to isolate reactions to unambiguous hate speech—the kind of language platforms themselves publicly cite as violations. For assessing cross-national preferences toward content moderation, such a standardized design ensured that respondents across diverse political and cultural contexts were reacting to the same clearly defined violation rather than to country-specific idioms or ambiguous cases. Our study offers strong internal validity for comparing global differences in moderation preferences. Future work at the national level could also examine content moderation preferences by varying targets that might be more relevant to their specific culture or context.

Respondents in our study appear to approach platform governance through a rights-based lens, emphasizing liberty and limits on authority rather than harm reduction or social cohesion. This value-based divide transcends national culture and political ideology, underscoring that support for, or resistance to, moderation is not merely situational: it reflects deeper normative worldviews about speech, harm, and the boundaries of democratic discourse.

Our study further reveals other meaningful variation. Most notably, the US and Sweden stand apart in content moderation preferences compared to the remaining countries. American and Swedish respondents are consistently less supportive of moderation, even when exposed to the kind of extreme language used in our treatment, though Americans show somewhat greater variability to who is being targeted than respondents in most other countries (support for hard moderation in the US varies from 47.5% in the gender treatment to 60.3% in the ethnic group treatment). These patterns echo the US’s unique legal and cultural commitment to liberty-centric models of free expression, and Sweden’s unusually expansive constitutional protections for speech (for European standards), which include some of the strongest guarantees of freedom of expression in the world.

While such commitments are central to the democratic traditions of both countries, they pose challenges in a global context, where platform governance increasingly must account for competing democratic values, such as dignity, equality, and harm reduction.

This divergence is especially consequential in the case of the US given that most of the world’s dominant social media platforms are headquartered there. This has significant implications. Our findings raise important questions about the extent to which US-centric values shape global content governance, particularly when these values diverge from public opinion in other democratic societies. While users around the world show strong support for moderation in the face of harmful speech, platform policies may not always reflect this consensus if they are calibrated to a narrower vision of free expression.

This matters because when platforms internalize the US’s liberty-centric model of speech, they risk misaligning with the cultural expectations and legal frameworks of the societies they serve elsewhere, thereby resulting in a legitimacy gap: users outside the US may perceive platforms as failing to adequately protect against harm, especially where local norms or democratic values prioritize dignity, equality, or inclusion. This mismatch can generate public backlash, trigger regulatory responses—as seen in the European Union’s DSA—or contribute to declining trust in platforms’ governance models. It may also lead to greater platform abandonment in some parts of the world than others. According to X’s 2025 DSA transparency report, the platform’s user base in the EU declined by more than 10.5 percent since its October 2024 report^48^, a drop considerably larger than the seven million users it was projected to lose in the US between April 2022 and April 2025^49^. While this European exodus may have been accelerated by Elon Musk’s vocal and provocative support for far-right groups across the continent, reporting from news outlets suggests that many users and organizations cited toxic rhetoric and weakened content moderation as key reasons for leaving.^50^ In short, the US’s outlier status is not just theoretical, it carries practical global consequences for how billions of people experience rights, risks, and participation in digital spaces. Future research could extend this work by examining behavioral responses to moderation environments, such as whether users would consider leaving platforms in the absence of content moderation.

As policymakers, civil society actors, and platform leaders grapple with how to balance speech rights with protections from harm, our findings offer the first global empirical foundation. They suggest that public attitudes toward content moderation are not as polarized or context-dependent as often assumed. At a time when online platforms are under growing pressure to both preserve open discourse and prevent digital harm, and also at a time when free speech is becoming extremely politicized, understanding how citizens across countries weigh these competing imperatives is essential for developing governance models that are democratically legitimate, culturally responsive, and globally coherent.

## Supplementary Information

Supplementary Material 1

## Data Availability

All data and code necessary to replicate this work will be made publicly available at https://osf.io/st2qp/.

## Footnotes

1 More information about quotas, weighting and details about the two attention checks we used can be found in the supplementary material A1.

2 Participants were informed before starting the survey about the sensitive nature of some of the items. They were given the option to discontinue at any point and they were fully debriefed at the end of the study. The research protocol was approved by the relevant ethics committee; further details on briefing and debriefing procedures are provided in the supplementary materials.

3 The vignette was presented as a stand-alone social media post, without contextual details such as the sender’s identity, replies, or platform-specific features, to maximize comparability across countries. This provides a clear and unambiguous example of hate speech that is comparable, even at some cost for ecological validity. By anchoring the stimulus in an example recognized by major platforms, the design ensures that cross-national differences in attitudes toward moderation reflect reactions to the same standardized violation rather than country-specific contexts or platform affordances.

4 We refer to “skin color” and “ethnic group” as a single category to ensure cross-cultural clarity. In some contexts, such as the US or UK, “race” is commonly used in public discourse, as well as in national surveys. However, in others—such as Germany or South Africa—the concept of race is considered inappropriate or legally sensitive, and references to ethnic origin are more socially and institutionally acceptable. The study received ethical approval from the University of Oxford’s research ethics committee (Ref No: SSH/DPIRC1A24006).

5 Note that the estimates come with uncertainty. The 95% confidence intervals for the pooled data look as follows: No Moderation: 6.7%, 7.5%, Soft moderation: 20.4%, 21.8% and Hard moderation: 70.9%, 72.5%. For the remainder of the study we will be reporting estimates only. The full results accompanied with 95% CIs can be found in Tables A1, A2 and A3 in the supplementary materials.

6 When estimating the probability of demanding each type of moderation using multinomial models, we find that the pairwise difference (i.e., the contrast in predicted probabilities) between Gender and Ethnic Group is statistically significant ($p = 0.006$), while the comparison between Gender and Sexual Orientation and the one between Sexual Orientation and Ethnic Group are not ($p = 0.066$ and $p = 0.148$, respectively). The estimates are derived from contrasting the predicted probabilities and the p-values are adjusted using the Tukey HSD method for multiple pairwise comparisons.

7 Figure A1 visualizes the statistical differences in moderation preferences across countries by type of moderation.

8 For full results and measures of uncertainty see Tables A2 and A3.

9 Accordingly, the items we employ are widely used to measure ideology in the largest survey projects, including the American National Election Study (ANES), the European Election Study (EES), the Australian Election Study (AES), the South African Social Attitudes Survey (SASAS), and the Brazilian Electoral Study (ESEB), covering all countries in our sample.

10 Parametric logit estimates of the relationship between ideology and hard moderation, which broadly confirm these patterns, are presented in Figure A2.

11 An interesting pattern appears in the case of France where, while ideology matters little for the *Gender* and *Sexual Orientation* targets, right wing respondents demand less moderation in the case of the *Ethnic Origin* group. In Sweden, the left-right gap is only evident in the *Gender* and *Ethnic Origin* groups. When exposed to the *Sexual Orientation* target, respondents across the left-right scale have high demands for hard moderation.

12 The parametric estimates of the relationship between free speech values and hard moderation confirm the general patterns and can be found in Figure A3.

13 The parametric equivalent for what appears on Fig. 2 can be found in the online supplementary materials (see Figure A5) and the 90-10% probability gaps appear in Table A5.
