# Supplementary material for: Ideology and free speech values predict content moderation preferences: cross-national evidence across targets of hate speech
Source: Sci Rep. 2026 Jun 17;16:19034. doi: 10.1038/s41598-026-56054-y (PMC13279812; doi:10.1038/s41598-026-56054-y)
Supplement: Supplementary file 1 — Supplementary Material 1 [file 41598_2026_56054_MOESM1_ESM.pdf]

Online Supplementary Materials

A1 Sampling . . . .
A2 Wording of Survey Items . . . .
A3 Additional Figures . . . .
A4 Tables . . . .
A5 Ethics & Compliance . . . .

A1 Sampling

The analysis in this paper is grounded in survey data collected from ten countries, chosen to reflect a diversity of cultural backgrounds, regulatory frameworks, and democratic traditions. The countries included are Australia, Brazil, France, Germany, Greece, Slovakia, South Africa, Sweden, the United Kingdom, and the United States. This geographical breadth allows for a comprehensive examination of public opinion on content moderation and freedom of expression across different societal contexts.

The survey's fieldwork was conducted between October 24 and November 26, 2024. The data collection was coordinated by the global polling firm Bilendi & Respondi in collaboration with the authors of this study, who were responsible for developing the survey instrument.

In each country, samples were constructed using nationally representative quotas to ensure demographic alignment with the general population. Specifically, quotas were applied for age, using five distinct categories; gender, with male and female categories; and educational attainment, which was classified into three levels according to the International Standard Classification of Education (ISCED). The total combined sample size for the survey is N=13,475. The analytic sample consists of those respondents who successfully passed two attention checks. The wording of the attention checks can be found in the next section of the supplementary material (A2).

After Bilendi & Respondi pre-screened survey responses for quality, we further excluded

ten respondents who failed the first attention check; none failed the second.

A2 Wording of Survey Items

Hateful posts (randomly assigned variations)

1. Imagine someone sends this social media post attacking a person because they have a different **skin color** or belong to a **different ethnic group** than the attacker: "they should be punished! We are not doing enough to rid us of those filthy animals" How should platforms (e.g., Facebook, Twitter/X, etc.) deal with this content?

2. Imagine someone sends this social media post attacking a person because they have a different **gender** to that of the attacker: "they should be punished! We are not doing enough to rid us of those filthy animals" How should platforms (e.g., Facebook, Twitter/X, etc.) deal with this content?

3. Imagine someone sends this social media post attacking a person because they have a different **sexual orientation** to that of the attacker: "they should be punished! We are not doing enough to rid us of those filthy animals" How should platforms (e.g., Facebook, Twitter/X, etc.) deal with this content?

Each respondent was asked either option 1, 2, or 3. The provided response options were:

- Leave it, do nothing
- Place a warning label on the post
- Reduce how many people can see the post
- Permanently remove the post
- Suspend the person's account

Key covariates

Free speech trade-offs. “In general, how important is freedom of speech relative to the harm it might cause?” (Anchors: (0) strongly prefer freedom of speech; (100) strongly prefer protection from harm)

Ideology. “In political matters people talk of ”the left” and ”the right”. How would you place your views on this scale?” (left (0) right (100))

Attention checks embedded in the survey

- Please indicate your agreement with the following statement. For our survey, it is essential that participants pay attention. To show us that you are reading this, please select both “Somewhat agree” and ”Strongly agree” here. (5-poind SA-SD response scale).
- When a big news story breaks, people often go to the media to check the details of what is going on. Our main aim, however, is to know if our respondents pay attention to our questions. Please ignore what we write in the question below and choose both Radio and Social media platforms. Based on the instructions above, what media do you normally choose? (Multiple-choice: Newspapers (Online); Radio; Television; The Internet; Social media platforms)

A3 Additional Figures

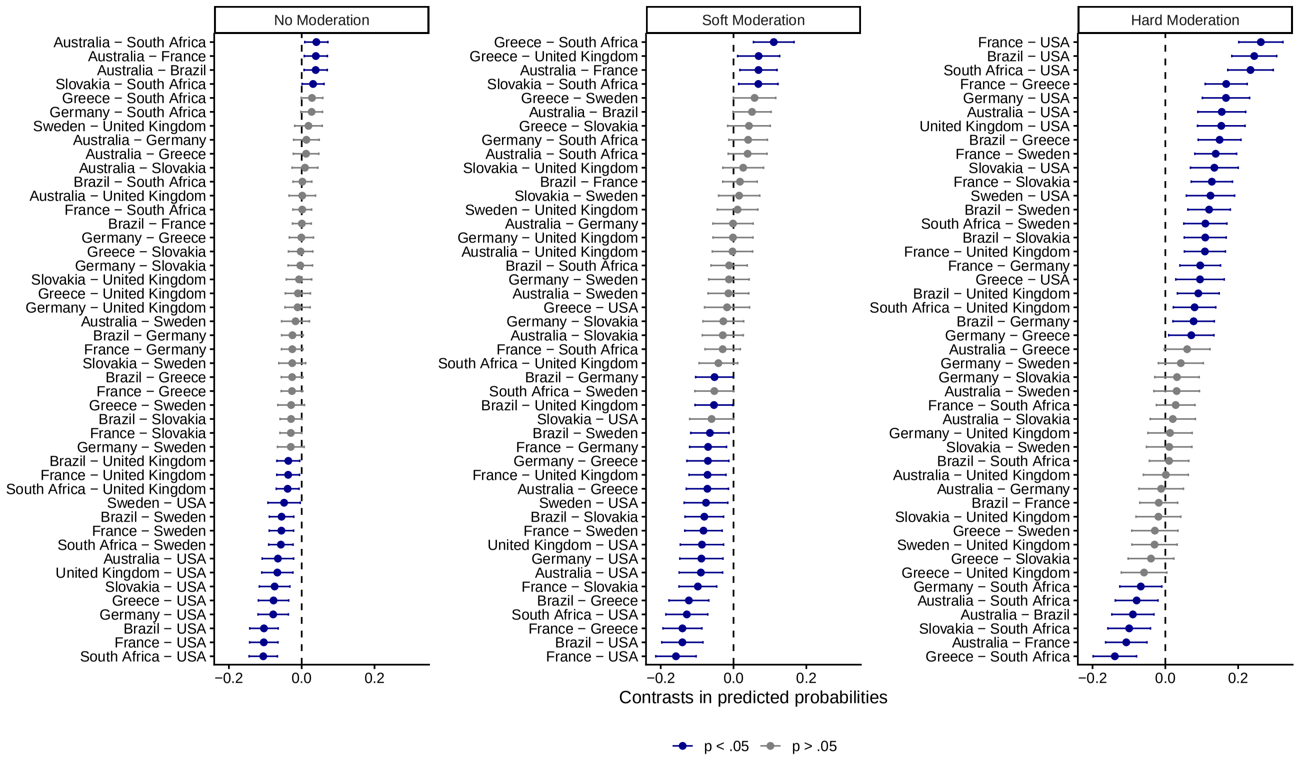

Figure A1: Contrasts and the corresponding 95% CIs in the predicted probability of preferring different types of content moderation across countries. Probability estimates are based on multinomial logit models.

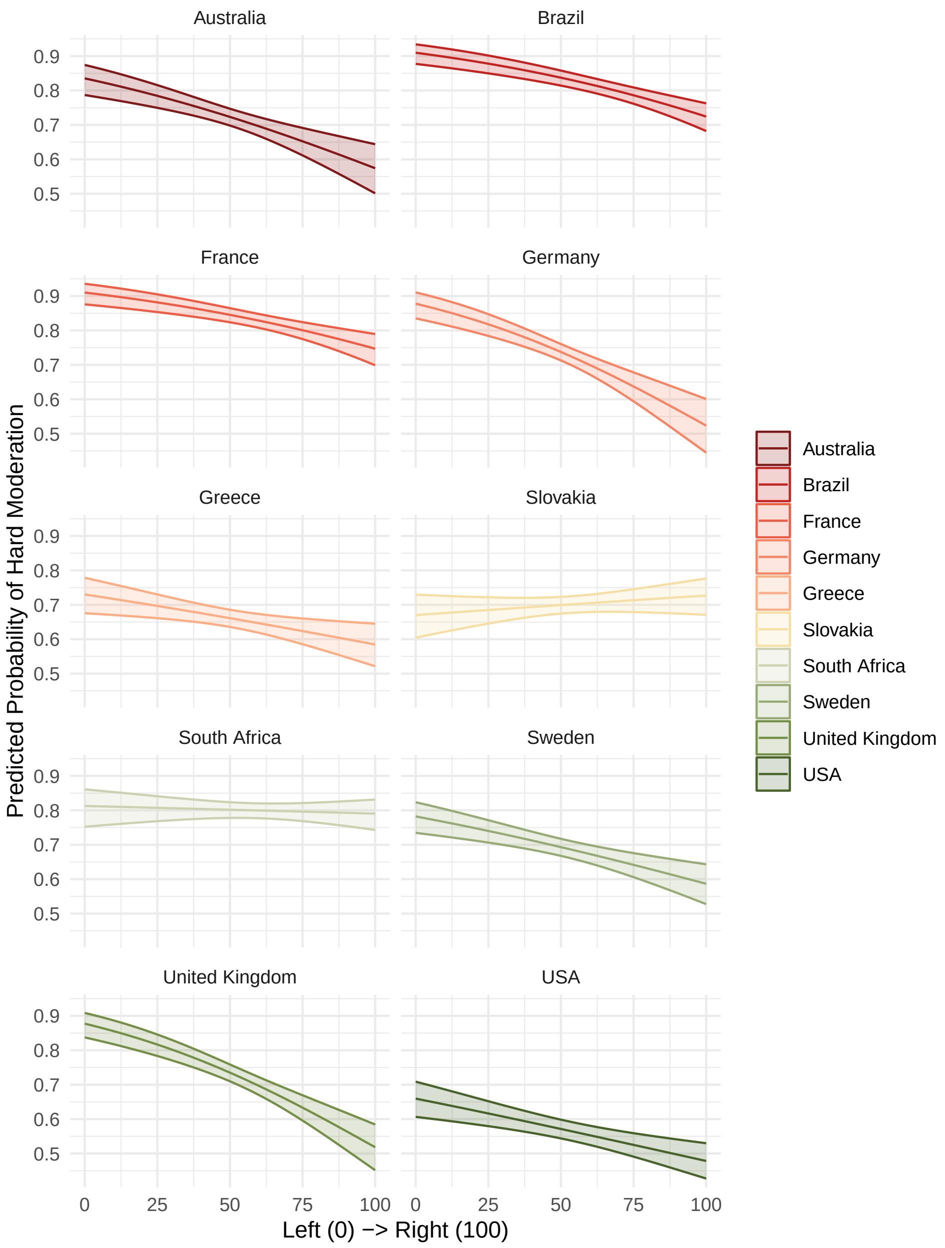

Figure A2: Predicted probability of preferring hard moderation across countries by Ideology. Probability estimates are based on logit models.

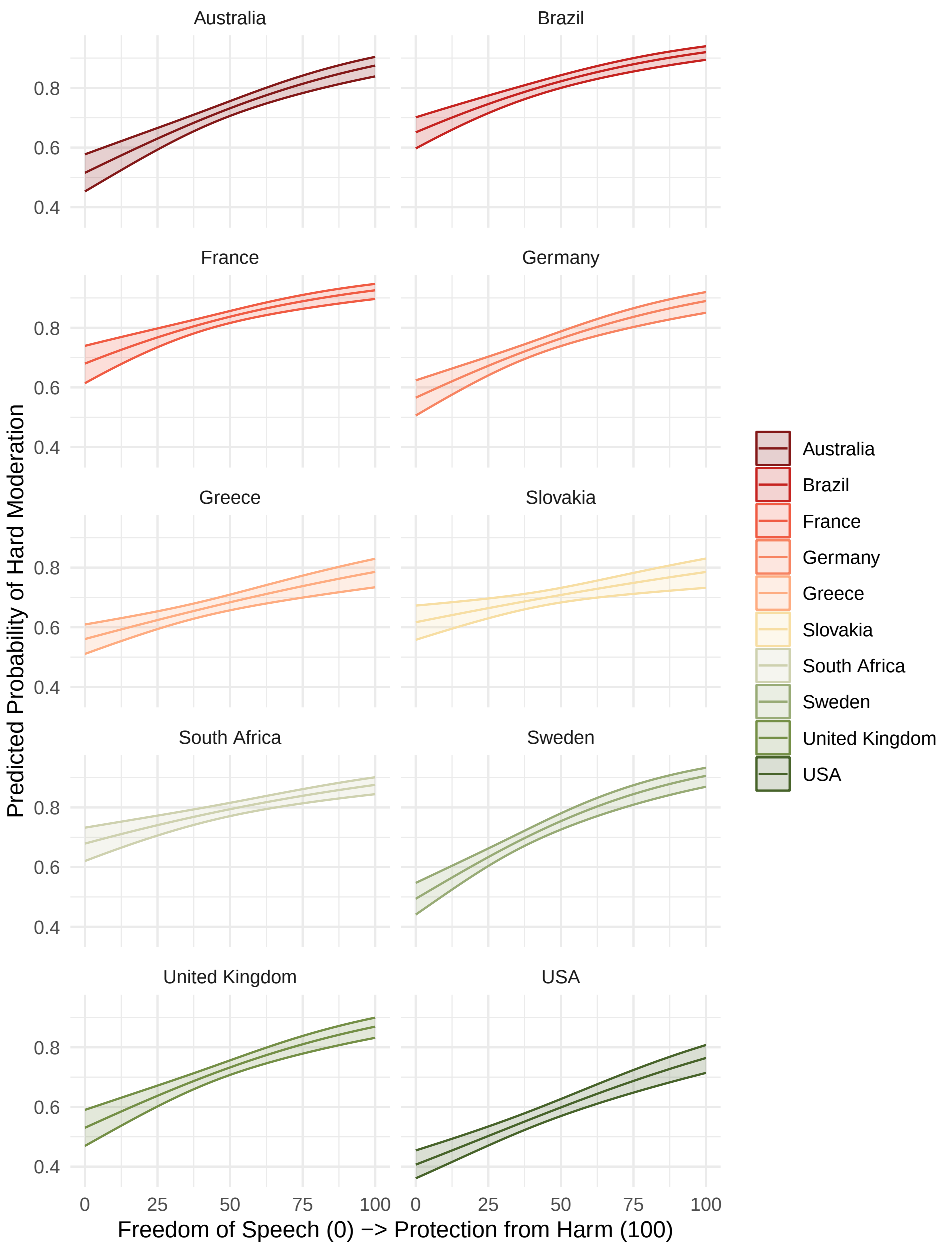

Figure A3: Predicted probability of preferring hard moderation across countries by Free speech values. Probability estimates are based on logit models.

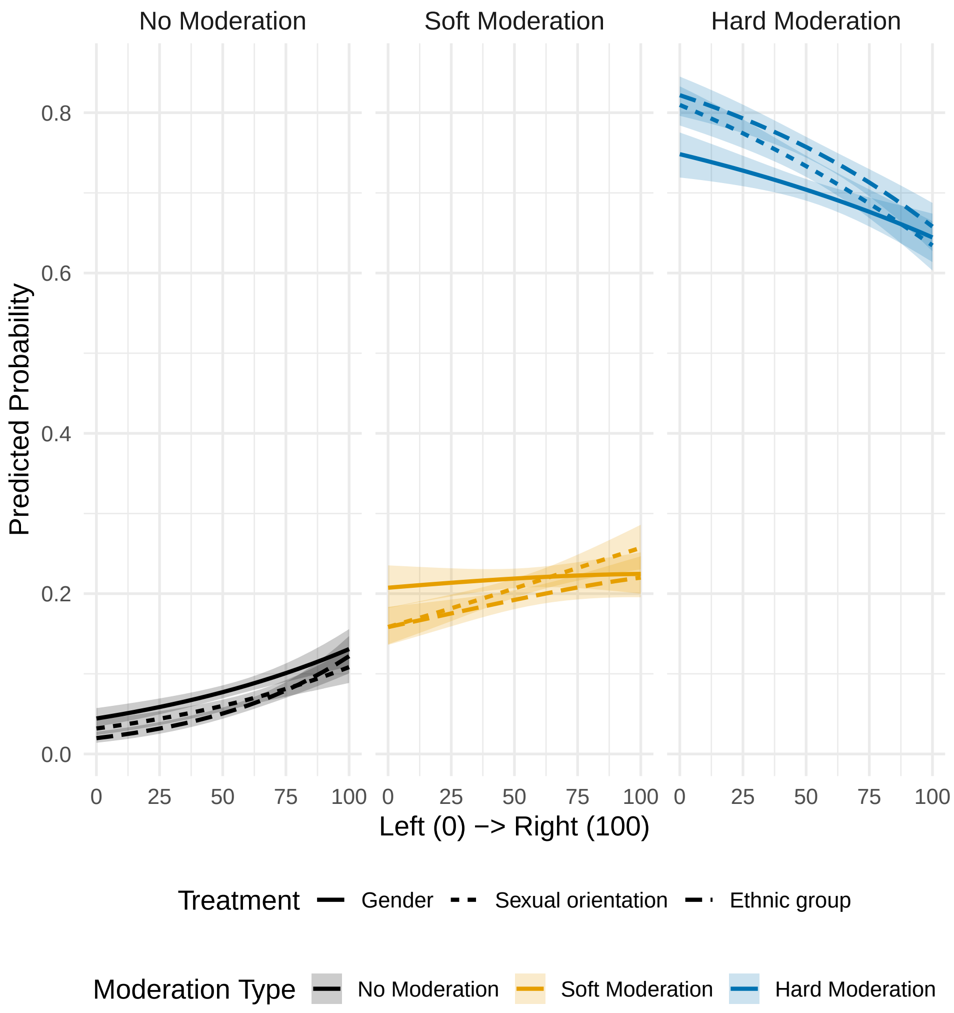

Figure A4: Predicted probabilities of preferring hard, soft, and no moderation across countries by Ideology. Probability estimates are based on a multinomial logit models.

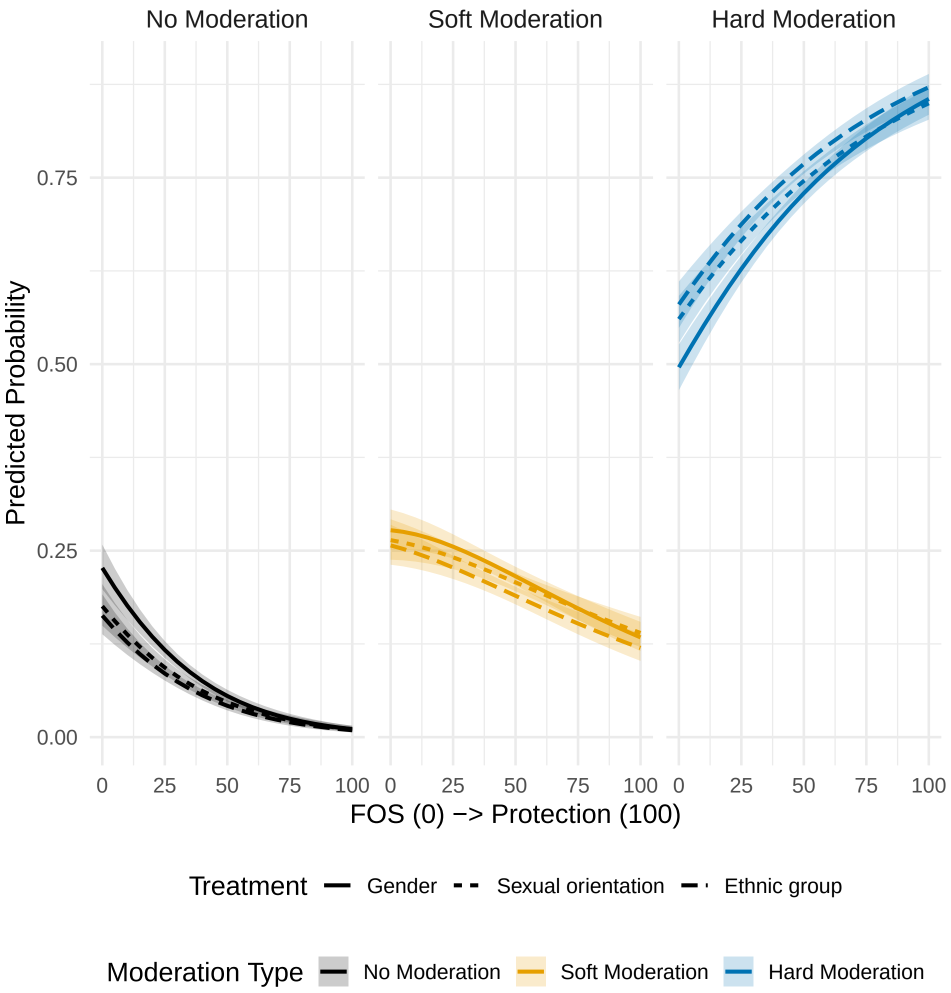

Figure A5: Predicted probabilities of preferring hard, soft, and no moderation across countries by FOS. Probability estimates are based on a multinomial logit models.

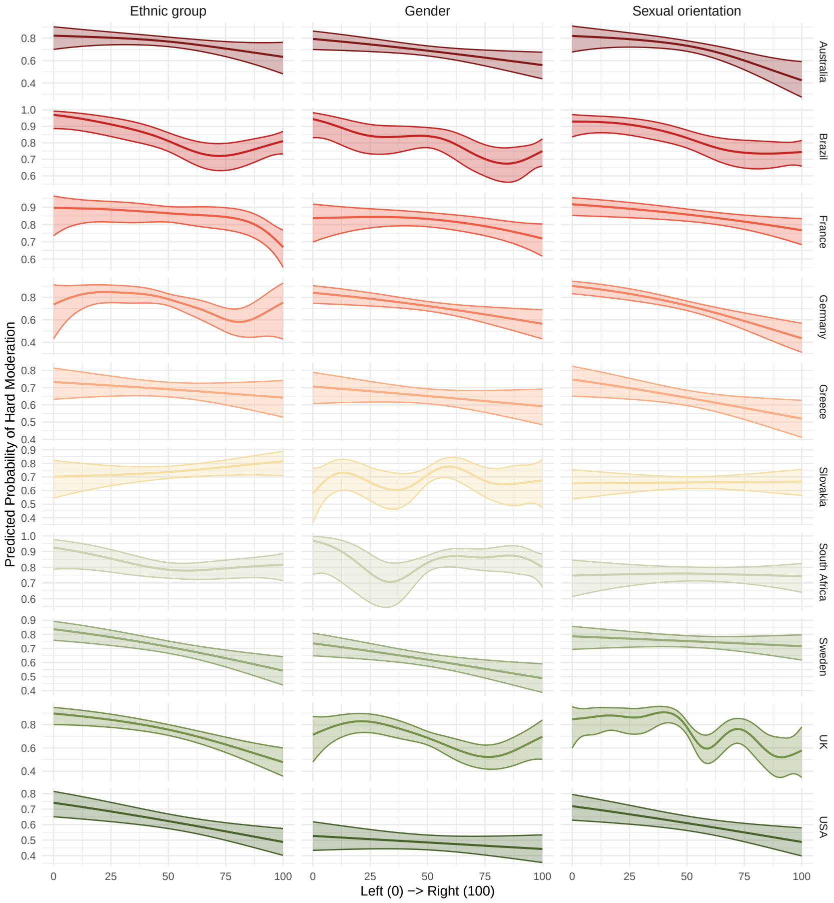

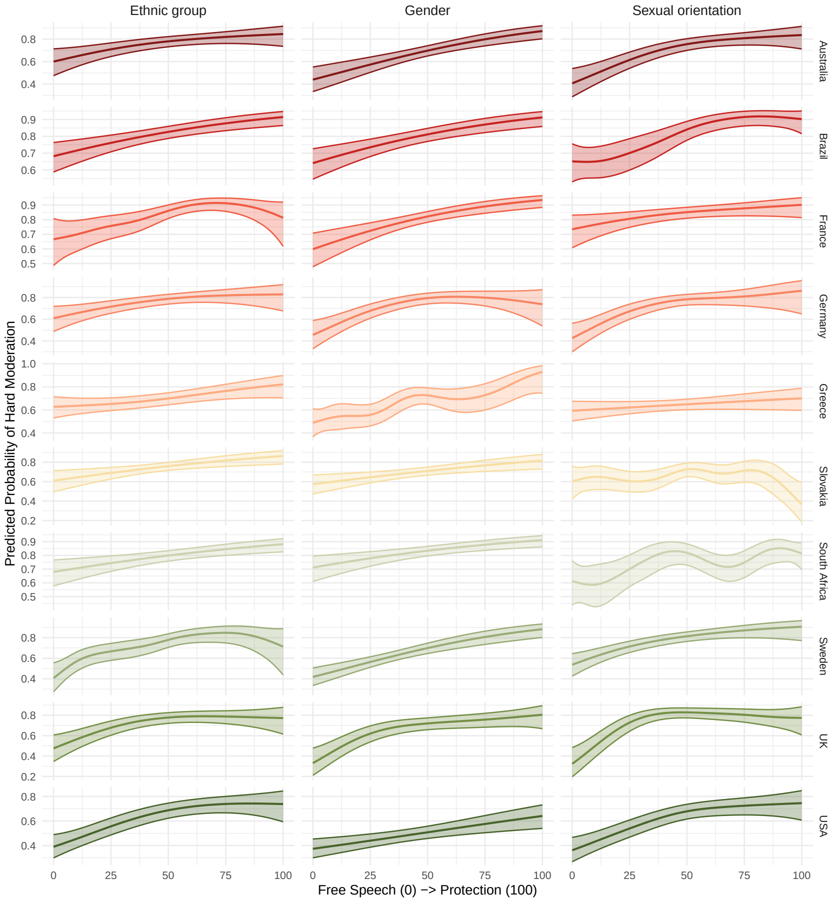

Figure A6: Predicted probabilities of preferring hard moderation across countries and treatment by left–right ideology (top) and free speech values (bottom). Estimates are smoothed using semiparametric Generalized Additive Models.

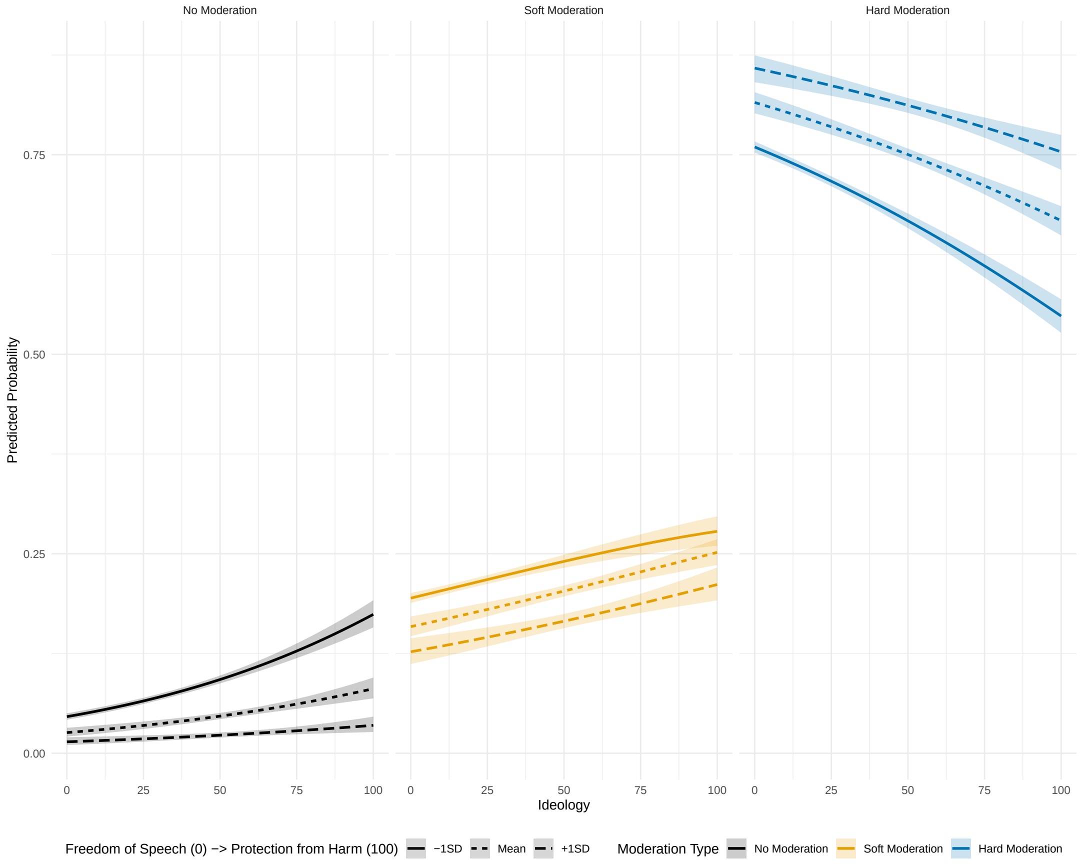

Figure A7: Predicted probabilities of preferring different types of moderation as a function of ideology and free speech values. Both graphs visualize the same equation $(Pr(Moderation_{ij}) = a + b_1 Ideology + b_2 FOS + b_3 Ideology \times FOS)$. The predicted probabilities are calculated across the left-right ideological placement, holding free speech at its mean value and varying it by ± 1 SD.

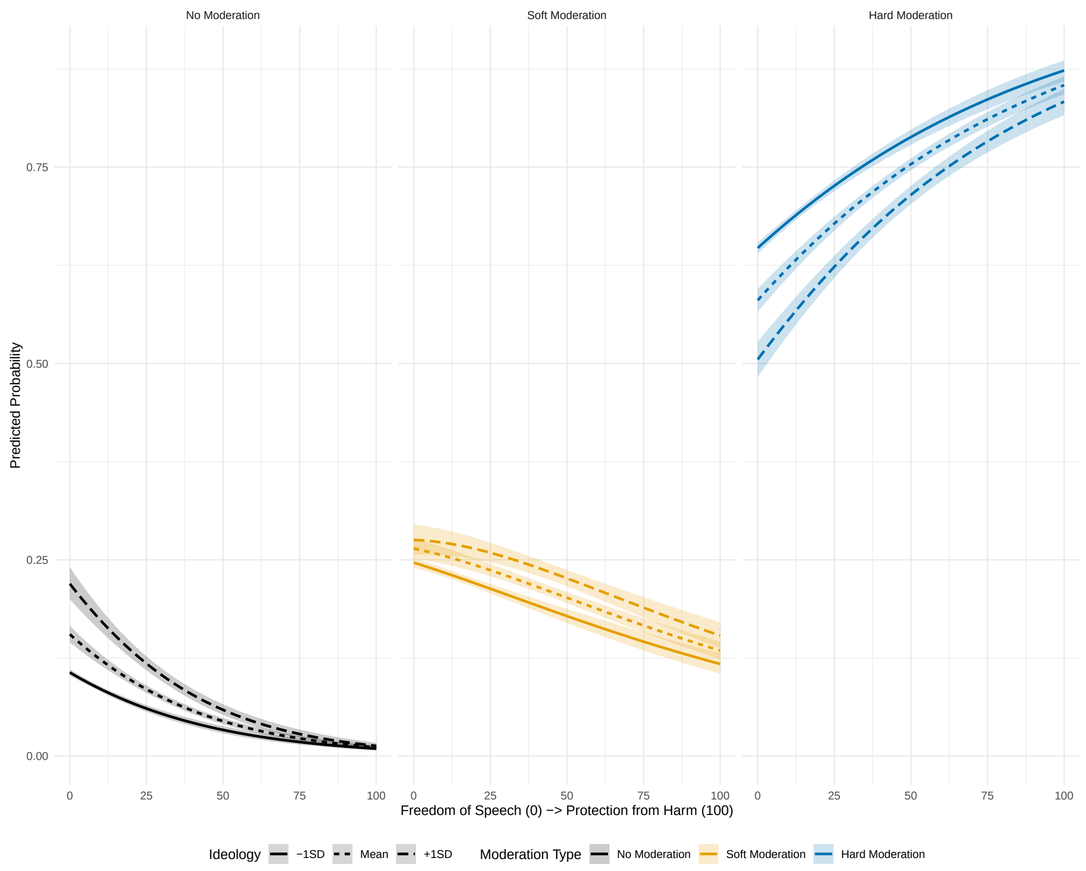

Figure A8: Predicted probabilities of preferring different types of moderation as a function of ideology and free speech values. Both graphs visualize the same equation $(Pr(Moderation_{ij}) = a + b_1 Ideology + b_2 FOS + b_3 Ideology \times FOS)$. The predicted probabilities are calculated across the free speech-protection from harm scale ideological placement, holding left right ideology at its mean value and varying it by ± 1 SD.

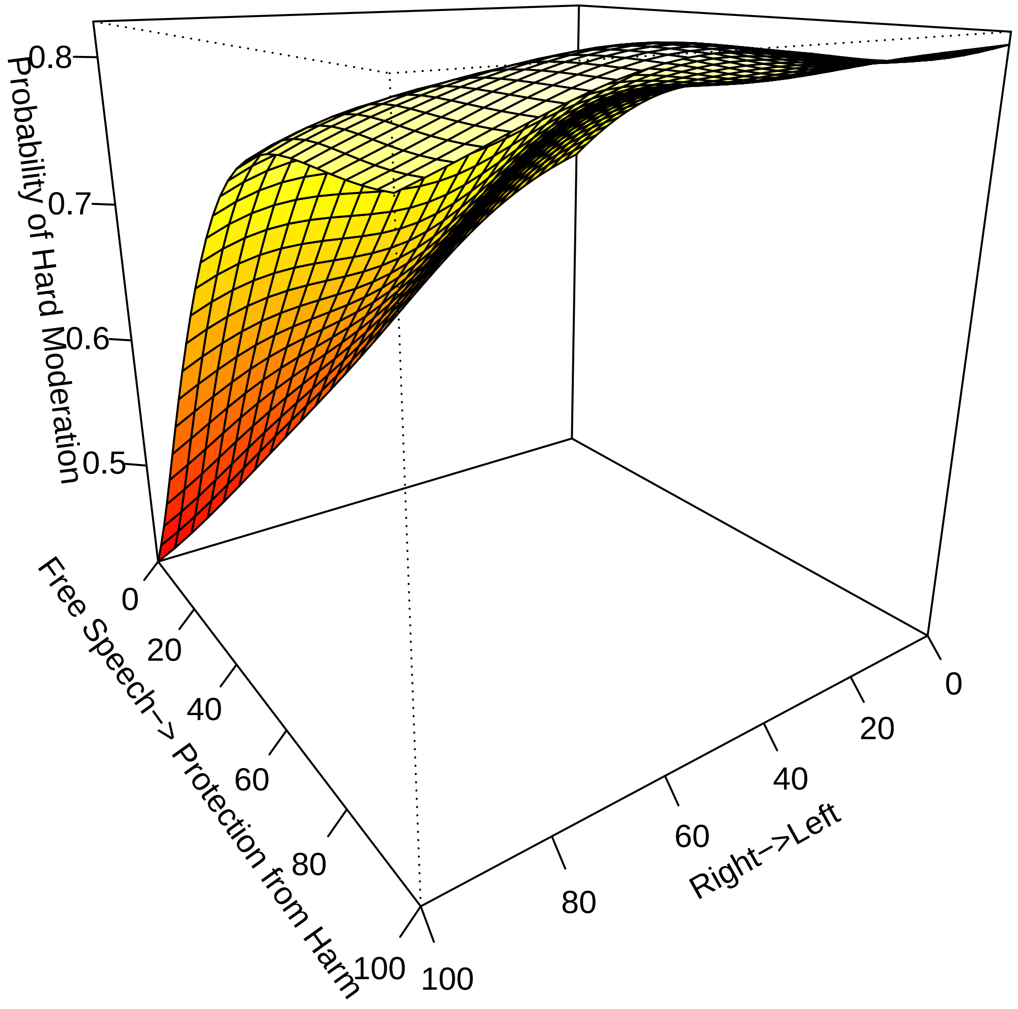

Figure A9: GAM model predicting hard moderation across values of left right placement and free speech values. The plane is averaged across individual country estimates of the interaction

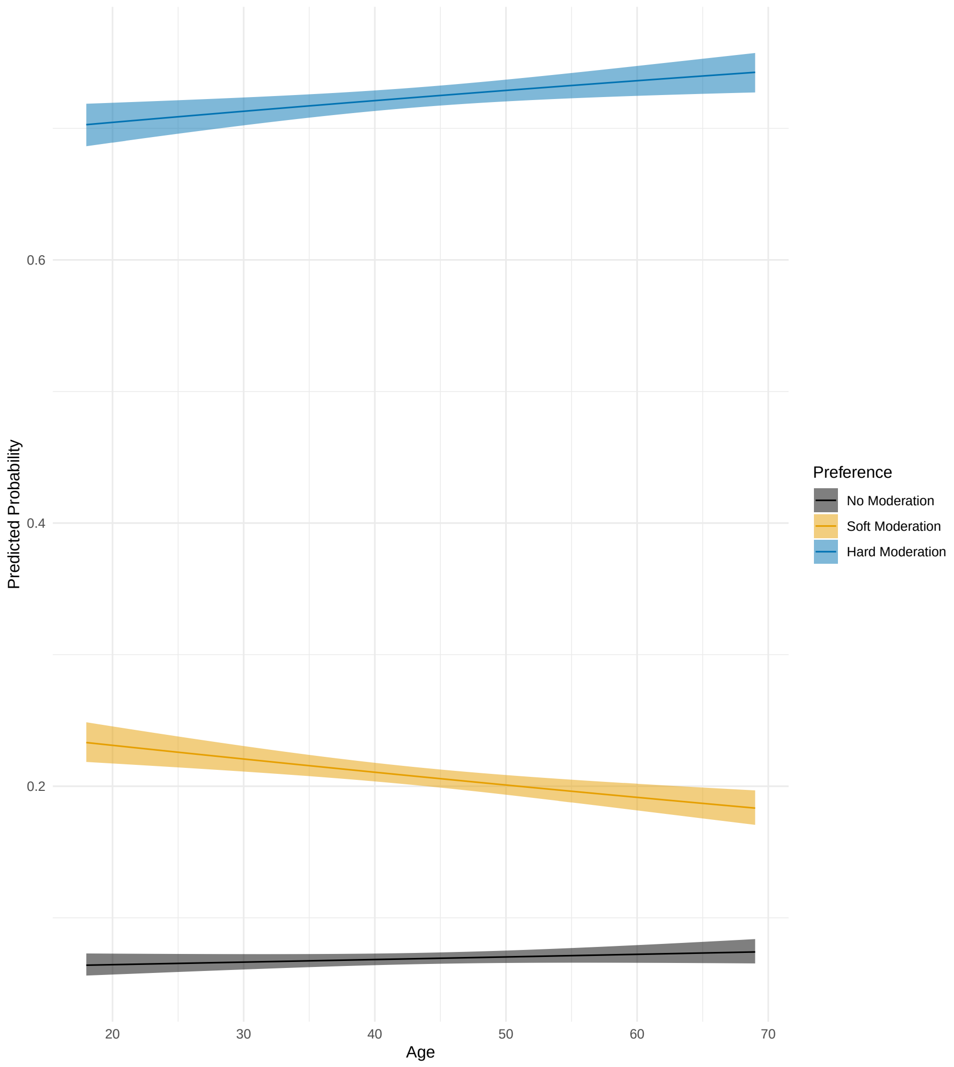

Figure A10: Predicted probabilities of moderation preferences across age groups. Estimates are derived from a multinomial model of the following form: $Pr(Moderation_{ij}) = a + b_1 Age + b_2 Treatment + b_3 Age \times Treatment$

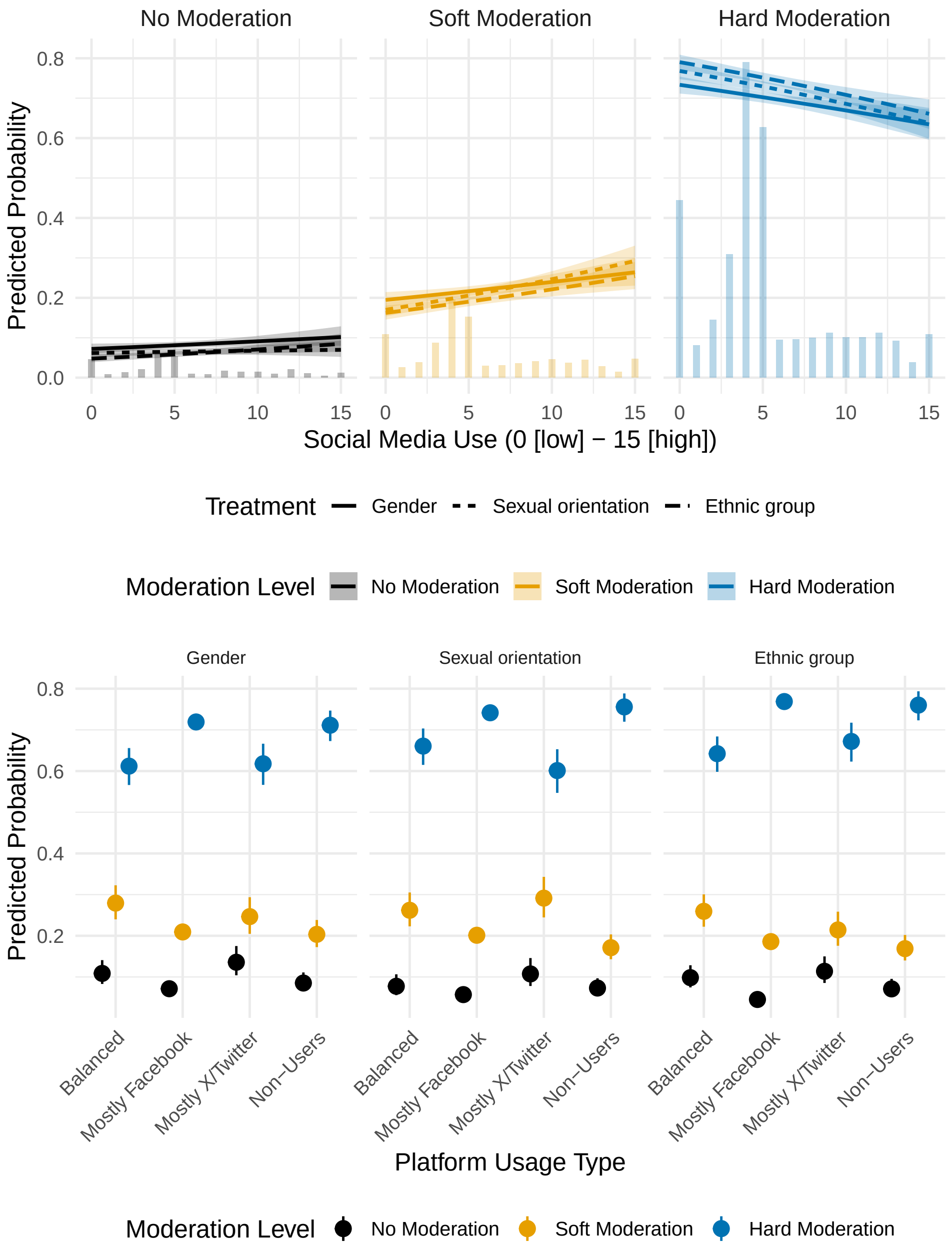

Figure A11: Predicted probabilities of moderation preferences by social media use (top panel) and by platform-usage type (bottom panel), based on a multinomial models.

A4 Tables

Table A1: Pooled distribution of moderation preferences across all countries

Moderation	%	CI Low	CI Upper
No Moderation	6.90	6.49	7.34
Soft Moderation	20.74	20.07	21.43
Hard Moderation	72.36	71.59	73.10

Table A2: Predicted moderation preferences across countries and targets with 95% confidence intervals

	Country	Target	Moderation	%	CI Low	CI Upper
1	Australia	Gender	No Moderation	10.41	7.44	13.39
2	Australia	Gender	Soft Moderation	21.39	17.39	25.39
3	Australia	Gender	Hard Moderation	68.20	63.65	72.74
4	Australia	Sexual orientation	No Moderation	8.66	5.99	11.33
5	Australia	Sexual orientation	Soft Moderation	21.17	17.29	25.06
6	Australia	Sexual orientation	Hard Moderation	70.17	65.82	74.52
7	Australia	Ethnic group	No Moderation	5.18	3.09	7.27
8	Australia	Ethnic group	Soft Moderation	19.66	15.92	23.41
9	Australia	Ethnic group	Hard Moderation	75.15	71.08	79.23
10	Brazil	Gender	No Moderation	5.20	3.07	7.33
11	Brazil	Gender	Soft Moderation	15.44	11.97	18.91
12	Brazil	Gender	Hard Moderation	79.36	75.47	83.24
13	Brazil	Sexual orientation	No Moderation	3.52	1.80	5.24
14	Brazil	Sexual orientation	Soft Moderation	14.81	11.49	18.12
15	Brazil	Sexual orientation	Hard Moderation	81.67	78.06	85.28
16	Brazil	Ethnic group	No Moderation	2.63	1.16	4.09
17	Brazil	Ethnic group	Soft Moderation	15.15	11.86	18.44
18	Brazil	Ethnic group	Hard Moderation	82.22	78.72	85.73
19	France	Gender	No Moderation	4.43	2.51	6.35
20	France	Gender	Soft Moderation	15.55	12.17	18.93
21	France	Gender	Hard Moderation	80.02	76.29	83.75
22	France	Sexual orientation	No Moderation	2.99	1.37	4.61
23	France	Sexual orientation	Soft Moderation	13.35	10.12	16.57
24	France	Sexual orientation	Hard Moderation	83.66	80.15	87.17
25	France	Ethnic group	No Moderation	4.75	2.81	6.69
26	France	Ethnic group	Soft Moderation	12.56	9.54	15.58
27	France	Ethnic group	Hard Moderation	82.69	79.24	86.13
28	Germany	Gender	No Moderation	6.97	4.56	9.38
29	Germany	Gender	Soft Moderation	21.05	17.19	24.92
30	Germany	Gender	Hard Moderation	71.97	67.72	76.23
31	Germany	Sexual orientation	No Moderation	6.91	4.54	9.27
32	Germany	Sexual orientation	Soft Moderation	20.98	17.18	24.77
33	Germany	Sexual orientation	Hard Moderation	72.12	67.94	76.30
34	Germany	Ethnic group	No Moderation	5.05	2.99	7.11
35	Germany	Ethnic group	Soft Moderation	19.38	15.66	23.09
36	Germany	Ethnic group	Hard Moderation	75.57	71.54	79.61
37	Greece	Gender	No Moderation	8.50	5.92	11.08
38	Greece	Gender	Soft Moderation	27.15	23.04	31.27
39	Greece	Gender	Hard Moderation	64.35	59.91	68.78
40	Greece	Sexual orientation	No Moderation	6.20	3.94	8.46
41	Greece	Sexual orientation	Soft Moderation	30.12	25.82	34.42
42	Greece	Sexual orientation	Hard Moderation	63.68	59.17	68.19
43	Greece	Ethnic group	No Moderation	5.22	3.16	7.29
44	Greece	Ethnic group	Soft Moderation	24.81	20.80	28.82
45	Greece	Ethnic group	Hard Moderation	69.97	65.71	74.22

	...continued Country	Target	Moderation	%	CI Low	CI Upper
46	Slovakia	Gender	No Moderation	6.90	4.59	9.22
47	Slovakia	Gender	Soft Moderation	24.05	20.15	27.95
48	Slovakia	Gender	Hard Moderation	69.05	64.83	73.26
49	Slovakia	Sexual orientation	No Moderation	8.52	5.93	11.11
50	Slovakia	Sexual orientation	Soft Moderation	26.01	21.94	30.08
51	Slovakia	Sexual orientation	Hard Moderation	65.47	61.06	69.89
52	Slovakia	Ethnic group	No Moderation	5.67	3.47	7.88
53	Slovakia	Ethnic group	Soft Moderation	20.36	16.52	24.20
54	Slovakia	Ethnic group	Hard Moderation	73.97	69.78	78.15
55	South Africa	Gender	No Moderation	3.06	1.45	4.67
56	South Africa	Gender	Soft Moderation	13.22	10.05	16.39
57	South Africa	Gender	Hard Moderation	83.72	80.27	87.18
58	South Africa	Sexual orientation	No Moderation	3.83	1.97	5.70
59	South Africa	Sexual orientation	Soft Moderation	20.62	16.68	24.55
60	South Africa	Sexual orientation	Hard Moderation	75.55	71.37	79.73
61	South Africa	Ethnic group	No Moderation	3.97	2.13	5.81
62	South Africa	Ethnic group	Soft Moderation	15.59	12.17	19.01
63	South Africa	Ethnic group	Hard Moderation	80.44	76.70	84.18
64	Sweden	Gender	No Moderation	11.85	8.86	14.84
65	Sweden	Gender	Soft Moderation	28.31	24.14	32.48
66	Sweden	Gender	Hard Moderation	59.84	55.30	64.37
67	Sweden	Sexual orientation	No Moderation	7.84	5.29	10.40
68	Sweden	Sexual orientation	Soft Moderation	20.41	16.58	24.24
69	Sweden	Sexual orientation	Hard Moderation	71.75	67.47	76.03
70	Sweden	Ethnic group	No Moderation	10.84	7.89	13.79
71	Sweden	Ethnic group	Soft Moderation	21.37	17.48	25.27
72	Sweden	Ethnic group	Hard Moderation	67.79	63.35	72.22
73	United Kingdom	Gender	No Moderation	9.42	6.73	12.12
74	United Kingdom	Gender	Soft Moderation	23.53	19.61	27.45
75	United Kingdom	Gender	Hard Moderation	67.04	62.70	71.39
76	United Kingdom	Sexual orientation	No Moderation	5.89	3.73	8.05
77	United Kingdom	Sexual orientation	Soft Moderation	19.35	15.72	22.97
78	United Kingdom	Sexual orientation	Hard Moderation	74.76	70.78	78.75
79	United Kingdom	Ethnic group	No Moderation	7.61	4.99	10.23
80	United Kingdom	Ethnic group	Soft Moderation	20.64	16.64	24.64
81	United Kingdom	Ethnic group	Hard Moderation	71.75	67.30	76.20
82	USA	Gender	No Moderation	18.06	14.33	21.80
83	USA	Gender	Soft Moderation	34.08	29.48	38.68
84	USA	Gender	Hard Moderation	47.86	43.01	52.71
85	USA	Sexual orientation	No Moderation	13.34	10.08	16.61
86	USA	Sexual orientation	Soft Moderation	26.90	22.65	31.16
87	USA	Sexual orientation	Hard Moderation	59.75	55.05	64.46
88	USA	Ethnic group	No Moderation	12.01	8.90	15.12
89	USA	Ethnic group	Soft Moderation	27.58	23.30	31.86
90	USA	Ethnic group	Hard Moderation	60.41	55.72	65.09

Table A3: Predicted moderation preferences across countries with 95% confidence intervals

	Country	Moderation	%	CI Low	CI Upper
1	Australia	No Moderation	8.03	6.53	9.53
2	Australia	Soft Moderation	20.73	18.49	22.96
3	Australia	Hard Moderation	71.25	68.75	73.74
4	Brazil	No Moderation	3.74	2.72	4.77
5	Brazil	Soft Moderation	15.13	13.19	17.06
6	Brazil	Hard Moderation	81.13	79.02	83.24
7	France	No Moderation	4.08	3.02	5.14
8	France	Soft Moderation	13.79	11.94	15.64
9	France	Hard Moderation	82.13	80.07	84.19
10	Germany	No Moderation	6.31	4.99	7.63
11	Germany	Soft Moderation	20.47	18.28	22.66
12	Germany	Hard Moderation	73.22	70.82	75.62
13	Greece	No Moderation	6.64	5.30	7.98
14	Greece	Soft Moderation	27.35	24.96	29.74
15	Greece	Hard Moderation	66.01	63.47	68.55
16	Slovakia	No Moderation	7.05	5.68	8.43
17	Slovakia	Soft Moderation	23.53	21.25	25.81
18	Slovakia	Hard Moderation	69.42	66.94	71.89
19	South Africa	No Moderation	3.61	2.59	4.64
20	South Africa	Soft Moderation	16.37	14.34	18.40
21	South Africa	Hard Moderation	80.01	77.82	82.21
22	Sweden	No Moderation	10.22	8.57	11.86
23	Sweden	Soft Moderation	23.47	21.17	25.77
24	Sweden	Hard Moderation	66.32	63.75	68.88
25	United Kingdom	No Moderation	7.63	6.19	9.07
26	United Kingdom	Soft Moderation	21.18	18.96	23.40
27	United Kingdom	Hard Moderation	71.19	68.73	73.65
28	USA	No Moderation	14.44	12.49	16.39
29	USA	Soft Moderation	29.49	26.95	32.02
30	USA	Hard Moderation	56.07	53.32	58.83

	Δ Pr	SE	CI Lower	CI Upper
Australia	-0.16	0.03	-0.23	-0.10
Brazil	-0.18	0.03	-0.23	-0.12
France	-0.14	0.03	-0.20	-0.08
Germany	-0.20	0.03	-0.26	-0.14
Greece	-0.10	0.03	-0.16	-0.03
Slovakia	0.04	0.04	-0.03	0.11
South Africa	-0.02	0.03	-0.08	0.05
Sweden	-0.14	0.03	-0.21	-0.07
United Kingdom	-0.24	0.03	-0.31	-0.17
USA	-0.16	0.04	-0.24	-0.08

Table A4: Differences in predicted probabilities of preferring hard moderation for extreme right and extreme left respondents. Note: Estimations are based on logit models. The 90th and 10th percentiles are calculated separately for each country.

	Δ Pr	SE	CI Lower	CI Upper
Australia	0.27	0.03	0.20	0.34
Brazil	0.27	0.03	0.20	0.33
France	0.16	0.03	0.11	0.21
Germany	0.22	0.03	0.16	0.29
Greece	0.18	0.04	0.11	0.26
Slovakia	0.11	0.03	0.05	0.18
South Africa	0.18	0.03	0.11	0.24
Sweden	0.31	0.03	0.24	0.37
United Kingdom	0.26	0.03	0.19	0.32
USA	0.31	0.04	0.24	0.38

Table A5: Differences in predicted probabilities of preferring hard moderation for respondents holding extreme protectionist or free speech values. Note: Estimations are based on logit models. The 90th and 10th percentiles are calculated separately for each country.

	DoF	RefDof	χ^2	pValue
Australia	3.09	3.84	23.91	0.00
Brazil	3.57	4.35	35.83	0.00
France	1.28	1.51	5.46	0.03
Germany	1.54	1.90	36.17	0.00
Greece	1.00	1.00	4.34	0.04
Slovakia	5.44	6.53	7.64	0.36
South Africa	3.54	4.32	4.27	0.40
Sweden	1.40	1.70	13.62	0.00
United Kingdom	3.69	4.55	54.33	0.00
USA	3.44	4.23	30.59	0.00

Table A6: Approximate significance of smooth terms when modeling the effect of ideology. In the context of GAMs, the effective degrees of freedom (DoF) indicate the flexibility of each smooth; values close to 1 suggest an approximately linear effect. The reference degrees of freedom (RefDof) are those used in the approximate χ^2 test of the null hypothesis that the smooth term has no effect. The reported p-values give the probability, under the null, of observing a test statistic at least as extreme as the one obtained.

	DoF	RefDof	χ^2	pValue
Australia	1.99	2.50	65.08	0.00
Brazil	4.48	5.43	67.94	0.00
France	2.31	2.89	43.81	0.00
Germany	2.49	3.11	60.95	0.00
Greece	1.04	1.08	37.92	0.00
Slovakia	2.12	2.65	14.50	0.00
South Africa	1.00	1.00	39.82	0.00
Sweden	2.13	2.67	92.03	0.00
United Kingdom	3.21	3.98	84.22	0.00
USA	2.51	3.11	121.90	0.00

Table A7: Approximate Significance of Smooth Terms when smoothing the freedom of speech effect. See Table above for interpretation of the DoF.

A5 Ethics & Compliance

We obtained ethical approval from the University of Oxford's Research Ethics Committee (approval number: SSH/DPIR_C1A_24_006). As this is a joint project with the Technical University of Munich (TUM), Oxford's approval covers all co-authors; TUM acknowledged this approval and required no separate review or waivers. The online survey complied with both universities' data-protection policies. The data was collected anonymously, without storing any direct identifiers, and is stored in both departments, where it is accessible to all co-authors. The data and the accompanying code will be made publicly available in an data repository for replication purposes.

All participants provided informed consent and were advised that some questions might be sensitive or cause distress. They were informed that participation was voluntary and that they could skip questions or withdraw from the study at any time without penalty. Contact information for concerns and the Oxford Research Ethics Committee's complaints procedure were provided. After the survey was completed, participants received an on-screen debriefing statement describing the purpose of the study and the nature of all the experimental stimuli.
